# A mutual activation loop between breast cancer cells and myeloid-derived suppressor cells facilitates spontaneous metastasis through IL-6 trans-signaling in a murine model

**DOI:** 10.1186/bcr3473

**Published:** 2013-09-10

**Authors:** Keunhee Oh, Ok-Young Lee, Suh Youn Shon, Onyou Nam, Po Mee Ryu, Myung Won Seo, Dong-Sup Lee

**Affiliations:** 1 Laboratory of Immunology and Cancer Biology, Department of Biomedical Sciences, Seoul National University College of Medicine, 103 Daehak-ro Jongno-gu, Seoul 110-799, Republic of Korea; 2 Interdisciplinary Program of Cancer Biology, Cancer Research Institute, Seoul National University College of Medicine, 103 Daehak-ro Jongno-gu, Seoul 110-799, Republic of Korea; 3 Transplantation Research Institute, Seoul National University College of Medicine, 103 Daehak-ro Jongno-gu, Seoul 110-799, Republic of Korea

**Keywords:** Myeloid-derived suppressor cell (MDSC), Breast cancer cell, Metastasis, IL-6 trans-signaling

## Abstract

**Introduction:**

Tumor cell interactions with the microenvironment, especially those of bone-marrow-derived myeloid cells, are important in various aspects of tumor metastasis. Myeloid-derived suppressor cells (MDSCs) have been suggested to constitute tumor-favoring microenvironments. In this study, we elucidated a novel mechanism by which the MDSCs can mediate spontaneous distant metastasis of breast cancer cells.

**Methods:**

Murine breast cancer cells, 4T1 and EMT6, were orthotopically grafted into the mammary fat pads of syngeneic BALB/c mice. CD11b^+^Gr-1^+ ^MDSCs in the spleen, liver, lung and primary tumor mass were analyzed. To evaluate the role of MDSCs in the distant metastasis, MDSCs were depleted or reconstituted in tumor-bearing mice. To evaluate whether MDSCs in the metastasizing tumor microenvironment affect breast cancer cell behavior, MDSCs and cancer cells were co-cultivated. To investigate the role of MDSCs in *in vivo *metastasis, we blocked the interactions between MDSCs and cancer cells.

**Results:**

Using a murine breast cancer cell model, we showed that murine breast cancer cells with high IL-6 expression recruited more MDSCs and that the metastasizing capacity of cancer cells paralleled MDSC recruitment in tumor-bearing mice. Metastasizing, but not non-metastasizing, tumor-derived factors induced MDSCs to increase IL-6 production and full activation of recruited MDSCs occurred in the primary tumor site and metastatic organ in the vicinity of metastasizing cancer cells, but not in lymphoid organs. In addition, tumor-expanded MDSCs expressed Adam-family proteases, which facilitated shedding of IL-6 receptor, thereby contributing to breast cancer cell invasiveness and distant metastasis through IL-6 trans-signaling. The critical role of IL-6 trans-signaling was confirmed in both the afferent and efferent pathways of metastasis.

**Conclusion:**

In this study, we showed that metastasizing cancer cells induced higher MDSCs infiltration and prompted them to secret exaggerated IL-6 as well as soluble IL-6Rα, which, in turn, triggered a persistent increase of pSTAT3 in tumor cells. This potential tumor-MDSC axis involving IL-6 trans-signaling directly affected breast cancer cell aggressiveness, leading to spontaneous metastasis.

## Introduction

Breast cancer is the leading cause of cancer-associated death in women worldwide [1]. Despite recent improvements in early detection and effective adjuvant chemotherapies, about one-third of patients with early disease will relapse with distant metastasis [2]. Metastasis of breast cancer remains a largely incurable disease and is the major cause of mortality among breast cancer patients [3]. Cancer metastasis is a complex process comprising dissociation of cancer cells from the bulk tumor, invasion of the neighboring tissue, intravasation, transport through the vascular system, extravasation, engraftment of disseminated cells and, finally, outgrowth of micrometastases [4]. In our previous study, orthotopically grafted human breast cancer cells expressing high levels of IL-6, but not those with low levels of IL-6, spontaneously metastasized to the lung and liver in immunocompromised NOD/scid/γ_c_-deficient (NOG) mice [5]. IL-6 signaling in cancer cells themselves imbued them with cancer stem cell properties and epithelial-to-mesenchymal transition (EMT) phenotypes, which facilitate cancer cell invasion into the surrounding tissue and blood vessels, and cause distant metastasis [5,6]. In addition, IL-6 is known to be an important mediator of the expansion and recruitment of myeloid-derived suppressor cells (MDSCs) [7,8].

MDSCs are a heterogeneous population of cells comprising immature cells of monocyte or granulocyte lineage. They expand dramatically under conditions such as trauma, tumor growth and various chronic inflammatory disorders, including infection, sepsis and immunization [7,8]. Originally described as suppressive myeloid cells, thus-expanded MDSCs negatively regulate immune responses through multiple contact-dependent and -independent pathways [8,9]. Nitrosylation of T cell receptors (TCRs) and CD8 molecules leads to defective cytotoxic T cell (CTL) responses, rendering the cells unresponsive to antigen-specific stimulation [10]. Shortage of L-arginine due to arginase I activity in MDSCs inhibits T cell proliferation by several mechanisms [11]. Nitrous oxide (NO) and transforming growth factor-β (TGF-β) produced by MDSCs induced further immunosuppressive microenvironments favoring tumor growth [7-9]. In addition to the abovementioned immunosuppressive functions, MDSCs actively formulate microenvironments favoring the generation and survival of cancer cells in association with chronic inflammation. Induced expression of IL-1β in gastric epithelial cells induces the recruitment of MDSCs and leads to gastric inflammation and cancer, while activation of nuclear factor-kappa B (NF-κB) in MDSCs is strongly associated with carcinogenesis [12]. MDSCs have been suggested to facilitate cancer cell metastasis through their immunosuppressive activities [8,13,14]. Recently, cancer-derived remote signals were shown to induce the accumulation of myeloid cells including MDSC populations in putative metastatic sites before migrating cancer cells arrived, forming a 'pre-metastatic niche', which aided extravasation of migrating cancer cells and facilitated new blood vessel formation [15-17]. Accumulating evidence shows that tumor-derived factors and tumor-cell-signaling mediators, such as Hsp72 and S1pr1, activate MDSCs to potentiate their immunosuppressive functions or increase the recruitment and colonization of these cells into pre-metastatic tissues [18,19]. Increased circulating MDSCs in breast cancer patients has been shown to be correlated with clinical cancer stage and metastatic tumor burden [20,21]. However, the evidence for the direct roles of cancer cell-exposed MDSCs in enhancing cancer cell aggressiveness, leading to spontaneous metastasis of these cells, from their invasion into the surrounding tissue and vascular system to their colonization of the target organ and the underlying mechanisms is either missing or merely circumstantial.

We questioned whether MDSCs activated by cancer cells directly increase breast cancer aggressiveness leading to spontaneous distant metastasis. To adequately evaluate the mutual interaction of breast cancer cells and inflammatory cells including MDSCs, we utilized murine models in which breast cancer cells were orthotopically grafted into immunocompetent syngeneic mice [22]. We found that murine breast cancer cells with high IL-6 expression recruited more MDSCs and that the metastasizing capacity of cancer cells paralleled MDSC recruitment in tumor-bearing mice. Depletion and addition of MDSCs from tumor-bearing mice, respectively, reduced and increased the distant metastasis of breast cancer cells. Metastasizing, but not non-metastasizing, cancer cells activated MDSCs, increasing their expression and secretion of both IL-6 and soluble IL-6Rα, and facilitated breast cancer cell invasiveness and distant metastasis through IL-6 trans-signaling, acting both in afferent and efferent metastatic pathways. Thus, we provide evidence that breast cancer cells and MDSCs formed a synergistic mutual feedback loop and that thus-potentiated MDSCs directly affect breast cancer cell aggressiveness, leading to spontaneous metastasis.

## Methods

### Animals

BALB/c mice were purchased from the Jackson Laboratory (Bar Harbor, MA, USA). Experiments involving mice were approved by the Institutional Animal Care and Use Committee of Seoul National University (authorization no. SNU05050203).

### Cell lines

The mouse breast carcinoma cell lines 4T1 (ATCC CRL-2539) and EMT6 (ATCC CRL-2755) were purchased from the American Type Culture Collection (Manassas, VA, USA) and maintained in RPMI 1640 (WelGENE, Daegu, South Korea) supplemented with 10% heat-inactivated fetal bovine serum (FBS) (GIBCO, Grand Island, NY, USA) and 1% antibiotics (100 U/ml penicillin and 100 μg/ml streptomycin) at 37°C in a humidified 5% CO_2 _atmosphere. IL-6-expressing EMT6 cells (EMT6_IL-6 cells) were established by transfection with the pcDNA3.1_IL-6 construct using PromoFectin (PromoKine, Heidelberg, Germany), according to the manufacturer's instructions. Control cells were transfected with the pcDNA3.1 vector only. Stably transfected clones were established by selection with G418 (Sigma-Aldrich, St. Louis, MO, USA) at a concentration of 500 μg/ml for three weeks. IL-6-expressing EMT6 (EMT6_IL-6) clones were selected by ELISA. IL-6-knockdown 4T1 cells (4T1_shIL-6 cells) and Stat3-knockdown 4T1 cells (4T1_shSTAT3 cells) were established using the lentiviral vectors containing the shRNA (Santa Cruz Biotechnology, Santa Cruz, CA, USA). Cells were infected with the shIL-6 or shSTAT3 virus and cultured in the presence of puromycin, and IL-6-knockdown 4T1 (4T1_shIL-6) and Stat3-knockdown 4T1 (4T1_shStat3) clones were selected by ELISA and Western blotting, respectively.

### Tumor models

4T1 and EMT6 cells (1×10^5^/mouse) were injected into the mammary fat pads of BALB/c mice. Tumor growth was monitored every other day thereafter. At 26 or 29 days after injection, the mice were euthanized and primary tumor masses, livers and lungs were fixed in 4% paraformaldehyde (PFA) for 24 hours and embedded in paraffin. Sections (5 μm) were stained with H & E for histopathological analysis. Numbers of tumor masses in the liver and lungs were determined under a dissecting microscope before fixing with 4% PFA. To deplete MDSCs, mice were intraperitoneally injected with 100 μg of anti-Gr-1 antibody (RB6-8C5, eBioscience, San Diego, CA) or control Rat immunoglobulin G 1 (IgG1) twice a week, starting three days after 4T1 cell injection. To block IL-6 trans-signaling in the afferent pathways of metastasis, 4T1 cells were injected into the mammary fat pads and gp130-Fc (R&D Systems, Minneapolis, MN, USA) was administered continuously using an osmotic mini-pump (5 or 10 μg for 14 days; Alzet, Cupertino, CA, USA). To block IL-6 trans-signaling in the efferent pathways of metastasis, 4T1 cells were injected intravenously into BALB/c mice and mice were intravenously injected with gp130-Fc (2.5 μg/mouse) 4 four days after cell injection.

### Flow cytometry and MDSC isolation

To analyze MDSCs, mice were sacrificed 19 or 21 days after cancer cell injection. Mononuclear cells from the liver, lungs and primary tumor masses were isolated by Percoll (Amersham, GE healthcare, Buckinghamshire, UK) gradient centrifugation. Tissues were digested with collagenase D (1 mg/ml, Invitrogen, Carlsbad, CA, USA) and DNase I (0.5 U/ml, Sigma-Aldrich) for one hour at 37°C. Cells were suspended in 30% Percoll, layered onto the top of a 70% Percoll gradient and centrifuged (800 × *g*, 20 minutes). The interface was retained. Next, the cells were incubated with mAbs to mouse CD11b, Ly6C and Gr-1 that were conjugated to phycoerythrin, PerCP-Cy5.5, or allophycocyanin (eBioscience). They were then analyzed using a FACSCalibur flow cytometer (BD Biosciences, San Jose, CA, USA) and FlowJo software (Tree Star, Ashland, OR, USA). To isolate splenic MDSCs from naïve or tumor-bearing mice, splenocytes were prepared and labeled with phycoerythrin-conjugated anti-CD11b mAb (BD Pharmingen, San Jose, CA, USA) and allophycocyanin-conjugated anti-Gr-1 mAb (eBioscience). CD11b^+^Gr-1^+ ^MDSCs were purified using a FACS Aria cell sorter (BD Biosciences). The purity after sorting was greater than 95%.

### Tumor and MDSC conditioned medium preparation

EMT6 cells (1×10^4^) and 4T1 cells (1×10^4^) were incubated for 72 hours on a 24-well plate and the culture supernatants were collected. To obtain MDSC CM, FACS-sorted splenic MDSCs (4×10^5^) were cultured for 24 hours. 4T1/MDSC-CM and EMT6/MDSC-CM were prepared by cultivating MDSCs (4×10^5^) in 50% (v/v) 4T1-CM or EMT6-CM for 24 hours. A volume of 4T1/MDSC-CM containing 1 ng of IL-6, or the same volume of EMT6/MDSC-CM or MDSC-CM, was added to the 4T1 and EMT6 cell cultures. To some cultures, the following signaling inhibitors were added; Stat3 inhibitor peptide (1 μM; Millipore, Billerica, MA, USA), PI3K inhibitor (LY294002, 10 μM; Calbiochem, Billerica, MA, USA), NF-κB inhibitor (Bay-117082, 10 μM; Calbiochem), JNK inhibitor (SP600125, 10 μM; Calbiochem), p38 MAPK inhibitor (SB403250, 10 μM; Cell Signaling, Danvers, MA, USA), and ERK inhibitor (PD98059, 10 μM; Calbiochem).

### Immunofluorescence microscopy

Tissues were fixed in 4% PFA and embedded in paraffin. Sections were stained with H & E for histopathological analysis. To investigate IL-6, IL-6Rα, and Adam17 expression levels in MDSCs, sections were stained with anti-Gr-1 mAb and other appropriate antibodies. The following primary antibodies were used: anti-mouse IL-6 (Abcam, Cambridge, MA, USA), anti-mouse IL-6Rα (Santa Cruz Biotechnology, Santa Cruz, CA, USA), anti-mouse Adam17 (Abcam) and anti-mouse Gr-1 (eBioscience). The following secondary antibodies were used: Alexa 488-conjugated anti-rabbit IgG (Invitrogen) and Alexa 594-conjugated anti-rat IgG (Invitrogen). Image acquisition and processing was performing using a confocal fluorescence microscope (Olympus, Center Valley, PA, USA) and an FV10-ASW 2.0 Viewer (Olympus).

### ELISA

EMT6 and 4T1 cells were plated on a 24-well plate (1 × 10^4^/well). The cells were permitted to grow for 24 or 48 hours. Supernatants were collected and assayed for IL-6 and soluble IL-6Rα levels by ELISA. For IL-6 detection, anti-mouse IL-6 (eBioscience) was used as the capture antibody, biotinylated anti-mouse IL-6 (eBioscience) in 0.1% BSA in PBS/T as the detection antibody and recombinant IL-6 (eBioscience) as the standard. To detect soluble IL-6Rα, we used anti-mouse IL-6Rα (R&D Systems) as the capture antibody, biotinylated anti-mouse IL-6Rα (R&D Systems) as the detection antibody and recombinant IL-6Rα (R&D Systems) as the standard.

### RNA analysis

RNA was isolated from sorted splenic MDSC using the RNeasy kit (QIAGEN; 74104, Hilden, Germany). cDNA was generated from 1 μg of total RNA by reverse transcriptase from Moloney Murine Leukemia Virus (M-MLV) (TAKARA, Shiga, Japan), and subjected to PCR. The following primer pairs were used for PCR: GAPDH, 5'-GTCAGTGGTGGACCTGACCT-3' and 5'-AGGGGTCTACATGGCAACTG-3'; IL-6, 5'-GACAAAGCCAGAGTCCTTCAGAG-3' and 5'-CTAGGTTTGCCGAGTAGATCTC-3'. PCR products were analyzed by 1.5% agarose gel electrophoresis.

### Western blot analysis

Cells were harvested in lysis solution containing 50 mM Tris/HCl (pH 7.6), 1% NP40, 150 mM NaCl, 2 mM EDTA, 100 μM PMSF, a protease inhibitor cocktail (Roche Applied Science, Basel, Switzerland), and a phosphatase inhibitor (Sigma-Aldrich). After incubation on ice for 30 minutes, cellular debris was removed by centrifugation (10 minutes, 4°C). Proteins (10 μg) were separated by SDS-PAGE and then transferred to a polyvinylidene difluoride membrane. After blocking with 5% skim milk, the membranes were probed with an appropriate antibody. Blots were developed with an enhanced chemiluminescence Western blotting detection system (Amersham, GE Healthcare, Buckinghamshire, UK). The following antibodies were used: anti-β-actin (Sigma-Aldrich), anti-phospho-Stat3 (Tyr705) (Cell Signaling, Danvers, MA, USA), anti-Stat3 (Santa Cruz Biotechnology), anti-I-κB (Santa Cruz Biotechnology), anti-phospho-JNK (Santa Cruz Biotechnology), anti-phospho-ERK (Santa Cruz Biotechnology), and anti-phospho-p38 (Cell Signaling).

### Invasion assay

Matrigel matrix solution (200 μg/ml, Matrigel™ Basement Membrane Matrix, BD Bioscience) was applied to each Transwell (Falcon, Franklin Lakes, NJ, USA). 4T1 cells (5×10^4^) were seeded into the upper chamber of the Transwell and then the lower chamber was filled with collagen matrix (5 μg/ml). Invasion assays were carried out for 18 hours. Non-invading cells on top of the matrix were removed by rubbing with a moistened cotton swab. Invaded cells on the lower surface of the Matrigel matrix were fixed with 4% PFA and stained with 0.2% crystal violet. Cells were counted using ImageJ software (version 1.46).

### Statistical analyses

The two-tailed Student's *t*-test was used to compare measurements for pairs of samples. Two-way analysis of variance (ANOVA) and Bonferroni *post hoc *testing were used to compare the tumor volumes of the two groups. The SPSS software (SPSS Inc.) was used for all statistical analyses.

## Results

### Correlation of spontaneous distant metastasis of breast cancer cells with MDSC recruitment

**Figure 1:**
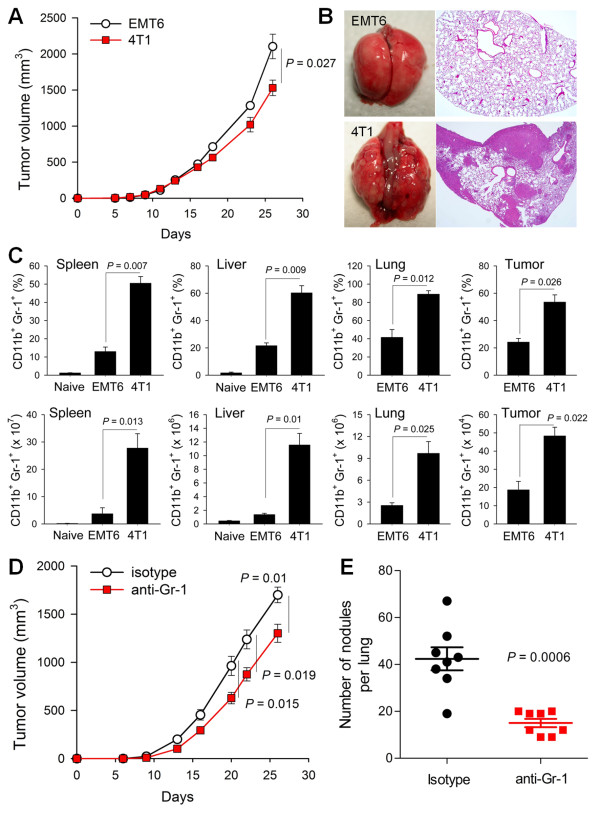
**Metastatic cancer cells facilitate recruitment of MDSCs**. **(A-B) **EMT6 and 4T1 cells were injected into the mammary fat pads of BALB/c mice. **(A) **Primary tumor growth (*n *= 8). **(B) **Representative photographs of lungs 26 days after cell injection (H & E). **(C) **The percentages and absolute numbers of MDSCs (CD11b^+^Gr-1^+^) at 19 days (*n *= 4). **(D) **4T1 cell-bearing mice were treated intraperitoneally with anti-Gr-1 antibodies. Primary tumor growth (*n *= 8). **(E) **Numbers of tumor nodules in the lungs from 4T1 cell-bearing mice at 26 days. MDSCs, myeloid-derived suppressor cells.

In our previous report, high IL-6-secreting human breast cancer cells revealed more aggressive phenotypes including enhanced distant metastasis [5] and recruited more inflammatory cells (our unpublished data) compared to the low IL-6 expressing cells. In another report, we also showed that damaged epithelial cells produced IL-6 and recruited inflammatory cells including neutrophils [23]. Thus, we assumed that IL-6 derived from cancer cells could affect the metastasis of cancer cells through inflammatory cell, including MDSC, recruitment. To elucidate the relationship between MDSC recruitment and distant metastasis of cancer cells, we created a murine breast cancer model using 4T1 and EMT6 breast cancer cells, which exhibit differential IL-6 expression [see Additional file 1, Figure S1]. 4T1 and EMT6 cells were orthotopically grafted into the mammary fat pads of syngeneic BALB/c mice. Primary tumor growth was slightly but significantly greater for EMT6 cells compared to 4T1 cells during the entire experimental period (Figure 1A). At 26 days after grafting, 4T1 cancer cells showed extensive lung metastasis, while EMT6 cancer cells showed no distant metastasis in the lung, liver, bone or brain (Figure 1B). IL-6-expressing 4T1 cell-bearing mice showed dramatic recruitment of CD11b^+^Gr-1^+ ^MDSCs in the spleen (a lymphoid organ), metastasizing organs (liver and lung) and primary tumor mass; the total number of MDSCs recruited was two to eight times higher in 4T1 cell-bearing mice than in EMT6 cell-bearing mice (Figure 1C and see Additional file 1, Figure S2). To further evaluate the role of MDSCs in the distant metastasis, the 4T1 cell tumor-bearing mice were depleted of MDSCs. Depletion of MDSCs reduced 4T1 lung metastasis (*P *= 0.0006) and primary tumor growth in the mammary fat pads (Figure 1D and 1E). These results show that MDSCs that expanded and recruited in the tumor-bearing mice are critically associated with the distant metastasis of cancer cells.

### Induction of IL-6 expression facilitated MDSC recruitment and increased their metastatic capacity

**Figure 2:**
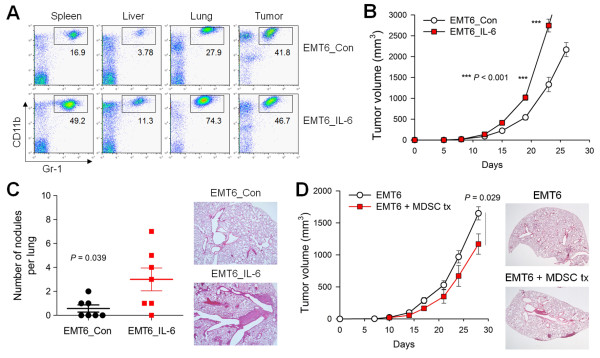
**Induction of recruitment and reconstitution of MDSCs in non-metastasizing EMT6 cell-bearing mice enhanced cancer cell metastasis**. **(A-C) **EMT6_Con and EMT6_IL-6 cells were injected into the mammary fat pads of BALB/c mice. MDSCs were analyzed at 21 days. **(A) **Percentages of MDSCs at 21 days (*n *= 6). **(B) **Primary tumor growth (*n *= 7). **(C) **Numbers of metastatic nodules in the lungs at 26 days (*n *= 7) and lung sections at 26 days (H & E). **(D) **EMT6 cells were injected into the mammary fat pads. Three days later, mice were intravenously injected with splenic MDSCs (5×10^6^/mouse) from 4T1 cell-bearing mice, a total of nine times. Primary tumor growth (*n *= 5) and representative photographs of lungs at 26 days. MDSCs, myeloid-derived suppressor cells.

We next evaluated whether IL-6-mediated MDSC recruitment promoted the metastasis of EMT6 cancer cells. We stably transfected EMT6 cells with a vector encoding murine IL-6 (EMT6_IL-6) [see Additional file 1, Figure S3A]. EMT6_IL-6 cancer cells grafted into the mammary fat pads of syngeneic recipients recruited more MDSCs to the spleen, liver, lung and primary tumor mass compared to the control empty vector-transfected EMT6 (EMT6_Con) cells (Figure 2A). The percentages and numbers of MDSCs recruited to these sites were comparable in EMT6_IL-6-bearing mice and 4T1 cell-bearing mice (Figure 1B and see Additional file 1, Figure S3B). EMT6_IL-6 cells showed increased tumor growth compared to the control EMT6_Con cells (Figure 2B). However, unexpectedly, distant lung metastasis was only slightly increased in EMT6_IL-6 cell-bearing mice (*P *= 0.039) (Figure 2C). Thus, we concluded that IL-6 secreted from breast cancer cells is an important and sufficient factor for MDSC expansion and recruitment, but that additional factors are required to facilitate the recruited MDSC-mediated metastasis of cancer cells.

To reconstitute a microenvironment that more closely resembles that of 4T1 cell-bearing mice, we adoptively transferred splenic MDSCs from 4T1 cell-bearing mice into EMT6 cell-bearing mice. MDSC-transferred EMT6 cell-bearing mice showed reduced primary tumor growth in the mammary fat pads, and only slightly increased lung metastasis, compared to vehicle-treated EMT6 cell-bearing mice (Figure 2D). Thus, neither repeated transfer of splenic MDSCs from metastatic tumor-bearing mice nor overexpression of IL-6 was sufficient to confer on non-metastasizing EMT6 cancer cells a metastasizing capacity comparable to that of 4T1 breast cancer cells. We assume that metastasizing cancer cells produce additional effects to potentiate the recruited MDSCs, thereby leading to distant metastasis.

### Metastasizing, but not non-metastasizing, breast cancer cells activated MDSCs

**Figure 3:**
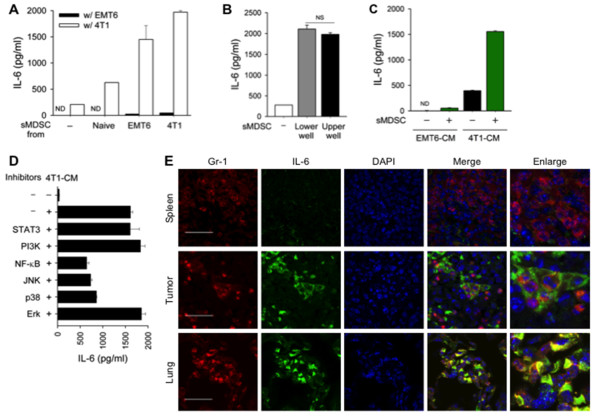
**Metastasizing cancer cell-derived factors induced excessive IL-6 production by MDSCs**. **(A) **Splenic MDSCs (4×10^5^) were co-cultured with 4T1 cells (1×10^4^) or EMT6 cells (1×10^4^) for 48 hours. IL-6 levels in the culture supernatants were measured by ELISA. **(B-C) **Splenic MDSCs from 4T1 cell-bearing mice were co-cultured with 4T1 cells in Transwell systems **(B) **or exposed to conditioned media (CM) for 24 hours **(C)**. **(D) **Splenic MDSCs were cultured with 4T1-CM in the presence of signaling inhibitors for 24 hours. **(E) **Immunofluorescence staining of Gr-1 (red), IL-6 (green), and DAPI (blue) in the spleen, tumors and lungs of 4T1 cell-bearing mice. Scale bar = 30 μm (original magnification, ×1,000). MDSCs, myeloid-derived suppressor cells.

To evaluate whether metastasizing, but not non-metastasizing, cancer cells further activate recruited MDSCs, we collected splenic MDSCs from naïve and tumor-bearing mice and co-cultivated them with 4T1 and EMT6 cells. Splenic MDSCs co-cultured with 4T1 cells showed increased production of IL-6, irrespective of their source (naïve, EMT6 cell-bearing, or 4T1 cell-bearing mice), compared to those co-cultured with EMT6 cells (Figure 3A). 4T1 cells co-cultured with splenic MDSCs provided activated signals either in the same chamber (lower) or a different chamber (upper) in a Transwell culture assay (Figure 3B), implying that contact-independent factors were important for activation of splenic MDSCs. To confirm the critical role of soluble factors derived from metastasizing breast cancer cells, conditioned media (CM) from breast cancer cells (4T1-CM and EMT6-CM) were applied to splenic MDSC cultures. 4T1-CM, but not EMT6-CM, enhanced the production of IL-6 by splenic MDSCs (Figure 3C). 4T1-CM increased IL-6 transcription in splenic MDSCs from both 4T1cell- and EMT6 cell-bearing mice; EMT6-CM and recombinant IL-6 only slightly induced the transcription of IL-6 [see Additional file 1 Figure S4]. Exposure of splenic MDSCs to 4T1-CM induced the activation of several signaling pathways, including Stat3, NF-κB, JNK, ERK and p38 pathways [see Additional file 1, Figure S5]. Using inhibitors of each pathway, we found that the NF-κB, JNK, and p38 signaling pathways were important in the production of IL-6 by activated MDSCs (Figure 3D). Importantly, confocal microscopic analysis of tissues from 4T1 cell-bearing mice revealed that MDSCs inside the primary tumor and lung strongly expressed IL-6 while those in spleen tissues from the same mice expressed little IL-6, but MDSCs in the primary tumor site of EMT6-bearing mice did not show increased expression of IL-6 (Figure 3E and see Additional file 1, Figure S6). In summary, metastasizing, but not non-metastasizing, tumor-derived factors induced MDSCs to produce more IL-6, and full activation of recruited MDSCs occurred in the primary tumor site and metastatic organs in the vicinity of metastasizing cancer cells.

### Activated MDSCs confer invasive potential on breast cancer cells and stimulate distant metastasis through IL-6 trans-signaling

**Figure 4:**
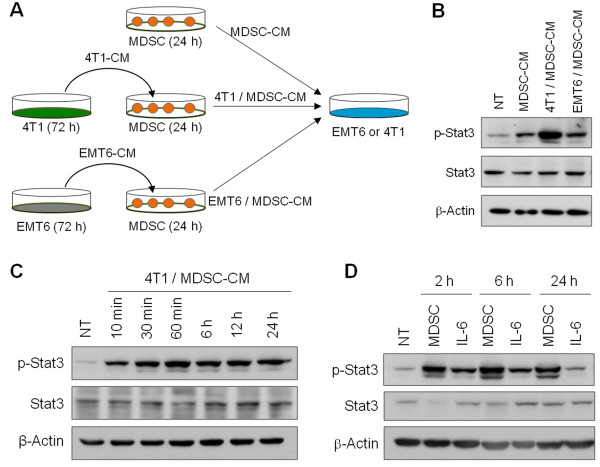
**Stimulation of MDSCs with metastasizing 4T1 cell-derived factors induced persistent Stat3 phosphorylation in cancer cells**. **(A) **Splenic MDSCs from 4T1 cell-bearing mice were cultivated in the presence of 4T1-CM or EMT6-CM. The conditioned media (MDSC-CM, 4T1/MDSC-CM, EMT6/MDSC-CM) were harvested and applied to 4T1 and EMT6 cancer cells. **(B-C) **Phospho-Stat3 and Stat3 levels in 4T1 cells exposed to each CM for 10 minutes (B) and for the indicated periods of time **(C)**. **(D) **4T1 cells (1×10^4^) were co-cultured with splenic MDSCs (4×10^5^) from 4T1 cell-bearing mice or recombinant mouse IL-6 (1 ng/ml). Phospho-Stat3 and Stat3 levels in 4T1 cells were determined by Western blotting after the removal of MDSCs. CM, conditioned media; MDSCs, myeloid-derived suppressor cells.

We next evaluated whether activated MDSCs in the metastasizing tumor microenvironment affect breast cancer cell behavior. We cultured 4T1 and EMT6 cells in CM from splenic MDSCs cultivated in the presence of 4T1-CM or EMT6-CM (4T1/MDSC-CM and EMT6/MDSC-CM, respectively) (Figure 4A). 4T1 cells cultured with splenic MDSC-CM showed mild phosphorylation of Stat3. Moreover, 4T1 cells cultured with 4T1/MDSC-CM, but not EMT6/MDSC-CM, showed greatly increased Stat3 phosphorylation within 10 minutes (Figure 4B). Stat3 phosphorylation levels were increased for 48 hours in 4T1 cells cultured in the presence of 4T1/MDSC-CM (Figure 4C and our unpublished data). Unlike 4T1/MDSC-CM, however, 4T1-CM did not induce the persistent activation of STAT3 [see Additional file 1, Figure S7]. Similar results were obtained for 4T1 cells co-cultured with splenic MDSCs, but not for 4T1 cells cultured in the presence of recombinant IL-6 (Figure 4D). These data suggest that IL-6 was important in inducing Stat3 phosphorylation in 4T1 cells, but that factors other than IL-6 from tumor-infiltrating MDSCs were needed for persistent Stat3 phosphorylation.

**Figure 5:**
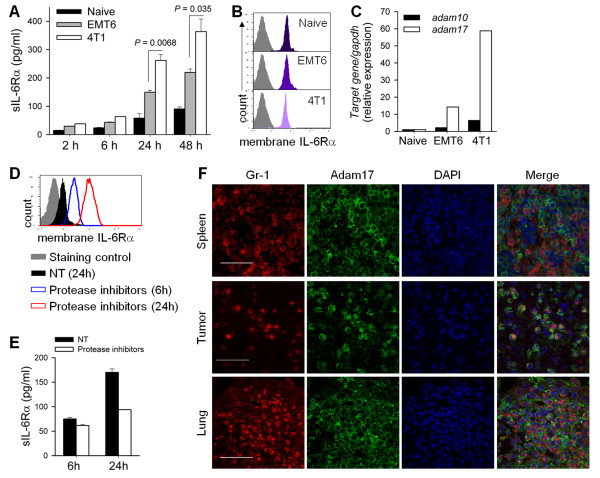
**Increased shedding of sIL-6Rα by the MDSCs of tumor-bearing mice**. **(A) **Soluble IL-6Rα levels in culture supernatants of splenic MDSCs were measured by ELISA and **(B) **surface IL-6Rα levels on splenic MDSCs were measured by FACS. **(C) **The mRNA expression of Adam10 and Adam17 in splenic MDSCs of naïve and tumor-bearing mice were determined by qRT-PCR. **(D-E) **Protease inhibitor cocktails were applied to cultures of splenic MDSCs from 4T1 cell-bearing mice for 6 or 24 hours. **(D) **Membrane-bound IL-6Rα was detected by FACS and **(E) **soluble IL-6Rα levels were measured by ELISA. **(F) **Tissue sections were stained for Adam17 (green), Gr-1 (red), and DAPI (blue) to compare their localizations. Scale bar = 30 μm (original magnification, ×1,000). FACS, fluorescence-activated cell sorting; MDSCs, myeloid-derived suppressor cells.

The recent characterization of IL-6 trans-signaling [24] suggests that tumor microenvironments may provide soluble IL-6Rα as well as IL-6 to maximally induce cancer cell aggressiveness through highly augmented IL-6 signaling, which is implicated in tumor cell survival, cancer stem cell characteristics and EMT phenotypes important for successful distant metastasis of cancer cells [5,6]. To investigate which cells in the tumor microenvironment provide soluble IL-6Rα, we measured levels of soluble IL-6Rα secreted from *ex vivo*-cultured splenic MDSCs from naïve, EMT6 cell-bearing, and 4T1 cell-bearing mice and 4T1 cancer cells. MDSCs from tumor-bearing mice generated more soluble IL-6Rα compared to 4T1 cells. Compared to those from naïve and EMT6 cell-bearing mice, splenic MDSCs from 4T1 cell-bearing mice produced more soluble IL-6Rα in *ex vivo *culture (Figure 5A and see Additional file 1, Figure S8). In contrast, splenic MDSCs from naïve, EMT6 cell-bearing and 4T1 cell-bearing mice expressed similar levels of surface IL-6Rα chain (Figure 5B).

Production of soluble IL-6Rα involves cell surface-associated proteases. Adam family proteases, especially Adam10 and Adam17, have been implicated in IL-6 trans-signaling [25,26]. Non-stimulated splenic MDSCs from 4T1 cell-bearing mice expressed increased levels of both Adam10 and Adam17 compared to MDSCs from EMT6 cell-bearing mice and naïve mice (Figure 5C). When we cultivated splenic MDSCs from 4T1 cell-bearing mice in the presence of protease inhibitors, levels of the membrane-bound form of IL-6Rα increased and those of soluble IL-6Rα levels decreased (Figure 5D and 5E). To further evaluate the critical role of ADAM family proteases in IL-6Rα shedding, we also utilized a more specific protease inhibitor, TAPI-2, an inhibitor of ADAM family proteases including ADAM17. TAPI-2, as well as a broad-spectrum protease inhibitors cocktail, decreased shedding of surface IL-6Rα. [see Additional file 1, Figure S9]. To confirm the expression of Adam17 and IL-6Rα by MDSCs *in vivo*, we analyzed spleen tissues, primary tumor masses and metastatic lesions in the lungs from 4T1 cell-bearing mice. Confocal microscopy showed that MDSCs in the spleen, primary tumor sites and lung expressed increased levels of Adam17 and IL-6Rα on their surfaces in 4T1 cell-bearing mice compared to those in EMT6 cell-bearing mice (Figure 5F and see Additional file 1, Figure S10). Thus MDSCs that were expanded and recruited in the metastasizing tumor-bearing mice were already capable of soluble IL-6Rα production, even in the spleen, a site remote from the metastasizing cancer cells. Taken together with the increased IL-6 levels only in the vicinity of metastasizing tumor cells, these findings suggest that IL-6 trans-signaling occurs preferentially in primary tumor sites and the metastatic lung but not in the spleen.

**Figure 6:**
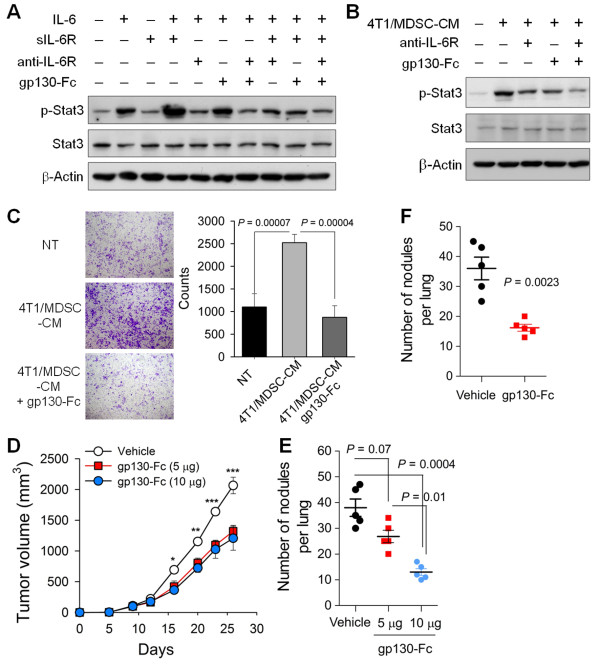
**Activated MDSCs contributed to tumor invasiveness through IL-6 trans-signaling**. **(A-B) **4T1 cells were treated with recombinant IL-6 plus soluble IL-6Rα **(A) **or 4T1/MDSC-CM **(B) **for 30 minutes in the presence of anti-IL-6R blocking antibody or gp130-Fc. **(C) **4T1 cells were allowed to invade through Matrigel for 18 hours in the presence or absence of 4T1/MDSC-CM and/or gp130-Fc (crystal violet). **(D-E) **4T1 cells were injected into the mammary fat pads. Some mice underwent continuous administration using osmotic mini-pumps (5 or 10 μg for 14 days). **(D) **Primary tumor growth and **(E) **numbers of metastatic masses in the lungs at 26 days. **(F) **4T1 cells were injected intravenously into BALB/c mice (*n *= 5 mice per group). Some mice received gp130-Fc (2.5 μg) 4 days after cancer cell injection. Numbers of metastatic masses in the lungs at day 12 were determined. Values are the means ± SEM of each group. *P < 0.05, **P < 0.01, ***P < 0.001. CM, conditioned media; MDSC, myeloid-derived suppressor cells.

To evaluate whether IL-6 trans-signaling is important for activation of 4T1 breast cancer cells, we cultivated 4T1 cells in the presence of IL-6 and/or soluble IL-6Rα and evaluated the individual and combined effects of a blocking anti-IL-6R antibody (which blocks both conventional IL-6 signaling and IL-6 trans-signaling) and a gp130-Fc fusion protein (which only blocks IL-6 trans-signaling). When applied individually, IL-6, but not soluble IL-6Rα, increased Stat3 phosphorylation in 4T1 cells. Treatment with both IL-6 and soluble IL-6Rα further increased the phosphorylation of Stat3, implying that IL-6 trans-signaling functioned in 4T1 cell activation (Figure 6A). Inhibition of IL-6 trans-signaling with gp130-Fc blocked Stat3 phosphorylation as efficiently as the IL-6R antibody (Figure 6A). To further confirm the role of IL-6 trans-signaling in the interaction of breast cancer cells and MDSCs, 4T1 cells were cultured in the presence of 4T1/MDSC-CM. gp130-Fc fusion protein treatment inhibited Stat3 phosphorylation in 4T1 cells to an extent comparable to IL-6R antibody treatment (Figure 6B). The enhanced IL-6 signaling mediated by the cancer cell-MDSC interaction augmented 4T1 breast cancer cell aggressiveness. 4T1 cells cultivated with 4T1/MDSC-CM showed exaggerated invasiveness in a Matrigel invasion assay, a response that was blocked by gp130-Fc treatment (Figure 6C). To investigate the role of IL-6 trans-signaling in *in vivo *metastasis, we administered gp130-Fc to the tumor-bearing mice. Continuous infusion of gp130-Fc (5 or 10 μg/ea/14 days), starting from the day following cancer cell injection, reduced primary tumor growth in the mammary fat pads (Figure 6D) and lung metastasis in a dose-dependent manner (Figure 6E). These findings support the critical role of IL-6 trans-signaling in breast cancer cell invasiveness and metastasis *in vivo*. As increased IL-6 trans-signaling in 4T1 cell-bearing mice was suggested to occur in the primary tumor sites and metastatic lung (Figure 3E), we evaluated whether increased IL-6 trans-signaling in the lung also affected the efferent phase of cancer cell metastasis. Treatment with gp130-Fc (2.5 μg/bolus, i.v.) on day 4 after intravenous cancer cell injection decreased the lung metastasis of 4T1 cancer cells compared to vehicle-treated controls (Figure 6F).

**Figure 7:**
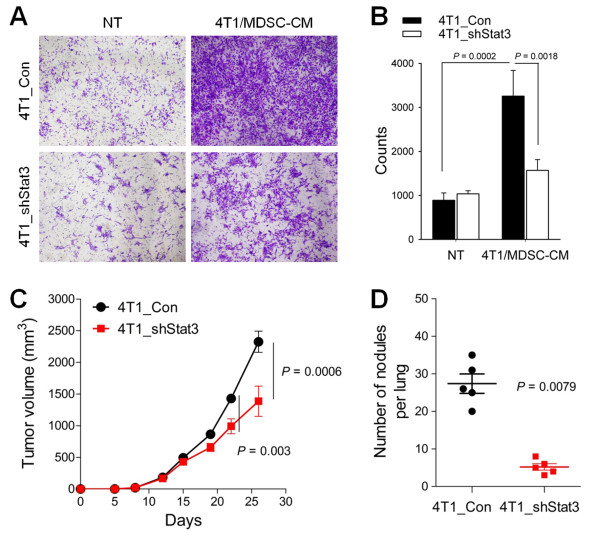
**Stat3 phosphorylation in MDSC-activated cancer cells contributed to tumor invasiveness and distant lung metastasis**. **(A-B) **4T1_Con and 4T1_shStat3 cells were allowed to invade through Matrigel for 18 hours in the presence or absence of 4T1/MDSC-CM. **(A) **Invaded 4T1 cells (crystal violet). **(B) **Invaded cells were counted using ImageJ software. **(C-D) **4T1_Con and 4T1_shStat3 cells were injected into the mammary fat pads. **(C) **Primary tumor growth. **(D) **Numbers of metastatic masses in the lungs at 26 days. Values are the means ± SEM of each group (*n *= 5). CM, conditioned media; MDSC, myeloid-derived suppressor cells.

Finally, to confirm whether the strong and persistent Stat3 phosphorylation in MDSC-potentiated cancer cells is crucial to spontaneous tumor metastasis, we generated Stat3-knockdown 4T1 (4T1_shStat3) cells [see Additional file 1, Figure S11]. 4T1_shSTAT3 cells revealed similar levels of IL-6 production and MDSC recruitments compared to 4T1_Con cells (data not shown). Greatly increased invasiveness in a Matrigel invasion assay was observed in control 4T1 (4T1_Con) cells, but not in 4T1_shStat3 cells, after treatment with 4T1/MDSC-CM, although reduced Stat3 expression itself had no effect on cancer cell invasiveness (Figure 7A and 7B). Primary tumor growth in the mammary fat pads was reduced in 4T1_shStat3 cell-bearing mice compared to 4T1_Con cell-bearing mice, while the reduction in distant lung metastasis was more dramatic in 4T1_shStat3 cell-bearing mice which exhibited few metastases (Figure 7C and 7D).

## Discussion

In this study, we showed that IL-6 derived from metastasizing murine breast cancer cells recruited MDSCs and tumor-expanded MDSCs expressed Adam-family proteases, which facilitated shedding of IL-6 receptors, thereby providing sIL-6Rα. In addition, factors other than IL-6, released from the cancer cells, promoted IL-6 production from recruited MDSCs in the vicinity of cancer cells. MDSC-derived IL-6 and sIL-6Rα induced persistent activation of STAT3 and increased invasiveness of breast cancer cells via an IL-6 trans-signaling mechanism. This IL-6 trans-signaling also increased distant metastasis *in vivo*. From these experiments, we provide novel information regarding potential tumor-MDSC synergistic axis involving IL-6 and soluble IL-6Rα [see Additional file 1, Figure S12].

MDSCs have been suggested to constitute tumor-favoring microenvironments largely through their suppressive effects on innate and adaptive immunity and promotion of angiogenesis [8,9,13,14]. In our murine breast cancer cell model, 4T1 breast cancer cells recruited more MDSCs and metastasized more strongly compared to EMT6 cells, not only in syngeneic immunocompetent BALB/c mice, but also in immunodeficient NOG mice, in which T, B, and NK cells are defective (our unpublished data and [27]). This implies that MDSCs in 4T1 cell-bearing mice induced spontaneous distant metastasis of cancer cells independently of their suppressive effects on adaptive and natural-killer cell anti-tumor immunity. Thus, in this study, we provide evidence that MDSCs potentiated by metastasizing breast cancer cells directly enhance the aggressiveness of cancer cells though trans-signaling by upregulating both IL-6 and sIL-6Rα secretion in primary tumor sites and the metastatic lung.

Induced expression of IL-6 in EMT6 cancer cells caused recruitment of MDSCs in lymphoid organs, metastatic target organs and primary tumor sites comparable to that caused by metastasizing 4T1 cancer cells, which implies that IL-6 or downstream signaling may be involved in this process. However, recruitment of MDSCs *per se *was not enough to guarantee that non-metastasizing breast cancer cells fully adopted metastatic capability. Transfer of splenic MDSCs from metastasizing 4T1 cell-bearing mice increased distant metastasis of non-metastasizing EMT6 cells but did not imbue them with the full metastatic capability of 4T1 cells. Based on the above findings, we assume that additional factors from metastasizing breast cancer cells affected the homing of MDSCs into the tumor sites and increased the potency of recruited MDSCs [18,19,28,29]. Our *in vitro *co-culture experiments showed that recruited MDSCs in the spleens of tumor-bearing mice required additional activation in the vicinity of metastasizing cancer cells, predominantly through contact-independent mechanisms. The outcome of activation of MDSC by metastasizing cancer cells *in vitro *can be summarized as exaggerated augmentation of IL-6 production by MDSCs [18,19]. Immunofluorescence microscopy of different tissues from 4T1 cell-bearing mice indeed showed that MDSCs in the primary tumor mass and metastatic lung, but not in the spleen, expressed high levels of IL-6. These findings suggest that recruited MDSCs may have different roles or function through different mechanisms depending on the recruited sites (lymphoid organs *versus *tumor sites, primary or metastatic) [30,31].

In contrast to the requirement for contact with metastasizing cancer cells for increased IL-6 production by MDSCs, the components necessary for increased soluble IL-6Rα production were increased in MDSCs in the remote sites of metastasizing tumor-bearing mice, but not those of non-metastasizing tumor-bearing mice. Expression levels of both IL-6Rα and the enzymes responsible for digesting the membrane form into soluble forms (Adam10 and Adam17) [25,26,32,33] were increased in the splenic MDSCs of 4T1 cell-bearing mice. Moreover, simple cultivation of splenic MDSCs from 4T1 cell-bearing mice increased the expression of soluble IL-6Rα compared to EMT6 cell-bearing mice. Thus, at least four remote signals were secreted by metastasizing 4T1 cancer cells; these induced (1) recruitment of MDSCs to various sites of tumor-bearing hosts, (2) increased expression of IL-6Rα, (3) increased expression of Adam family proteases, and (4) highly increased expression of IL-6 by MDSCs [34,35,18,19,29]. Further studies are needed to clarify the critical roles of the various mediators that may be involved in MDSC modulation. In this study, we convincingly demonstrated that 4T1 cells responded to IL-6 trans-signaling by MDSCs. However, as we did not perform experiments specifically inhibiting expression of IL-6 and sIL-6Rα in MDSCs *in vivo*, we cannot absolutely rule out the possibilities that IL-6 and sIL-6Rα responsible for metastasis could potentially be coming from other cell types *in vivo *either the tumor cells themselves or other cells within the tumor microenvironment. Further studies are needed to clarify this aspect.

The importance of IL-6 signaling in promoting tumorigenesis is well-documented, particularly for tumors associated with chronic inflammation such as colitis-associated colon cancer, pancreatic cancer and hepatocellular carcinoma [36-39]. In addition to these tumors, increased IL-6 signaling is preferentially found in basal-like breast cancers and high-grade tumors and is associated with a poor response to chemotherapy, increased distant metastasis in xenograft animal models and decreased metastasis-free survival in patients [5,6]. Thus, IL-6 signaling has been linked to tumor aggressiveness, including cancer stem cell phenotypes [5,6,40,41] and EMT phenotypes [42], drug resistance [43], and anoikis resistance, that is, contact-independent survival, which is required for travel through the vascular system [44]. In addition to tumor-derived IL-6 autocrine signaling [5,6], paracrine IL-6 signaling within tumor microenvironments has been highlighted recently. Mesenchymal stem cells constitute the cancer stem cell niche by providing IL-6 and CXCL7 [45,46]. Paracrine IL-6 signaling from tumor-infiltrating inflammatory cells is more important because these cells have a greater inflammatory cytokine secretion capacity, including IL-6 [47-49]. The caveat for this paracrine signaling is that cancer cells should express sufficient receptor machineries to recognize the increased IL-6 supply from the microenvironments. In this way, aggressive breast cancer cells exploited a trans-signaling mechanism by inducing the expression of molecules responsible for production of soluble IL-6 receptors (Adam proteases) from the recruited inflammatory cells [50]. In terms of STAT3 signaling as a downstream of IL-6, previous reports suggested that knocking down STAT3 using the shRNA technique in 4T1 cells leads to decreased invasiveness of the cells *in vitro *[51]. The differences between this report and our current study may lie in the perfectness of STAT3 knockdown considering the total absence of phosphorylated STAT3 in the previous report compared to our current study (our unpublished data).

The site of MDSC function in the metastatic tumor-bearing mice requires further comment. In terms of sites of MDSC immunosuppressive activity, the available data are contradictory. Some authors suggest that lymphoid organs, including the liver, are the primary sites of MDSC accumulation and immunosuppression [30,52], while others emphasized effector sites, such as inflammatory sites and tumors, but not lymphoid organs, such as the spleen [31,53,29]. In terms of MDSC function during the efferent phase of tumor metastasis and related angiogenesis, accumulation of MDSCs in the lung, a metastatic target organ, supported the effective engraftment of metastatic tumor cells at this site [15,19,54]. Because the metastasis-promoting effects of MDSCs in this study occurred in the absence of adaptive immunity and natural-killer cell activity and there was no increase in IL-6 signaling in the spleen, MDSCs themselves must have directly increased tumor cell metastatic capability in the tumor sites, either primary tumors or metastases, but not in the lymphoid organs, affecting both the afferent and efferent phases of metastasis through exaggerated IL-6 trans-signaling. Lastly, although we clearly showed the direct effects of MDSCs on the *in vitro *invasiveness and effector phase of lung metastasis, the co-occurrence of reduction in the primary tumor size and metastasis in some experiments prevented us to totally rule out the possibilities that differences in metastasis might be due to differences in tumor size rather than specific effects of MDSCs on metastatic capacity.

## Conclusions

Our findings reveal that breast cancer cells and MDSCs form a synergistic mutual feedback loop and that thus-potentiated MDSCs directly affect breast cancer cell aggressiveness, leading to spontaneous metastasis. We have shown that more MDSCs were recruited to the different organs of mice implanted in the mammary fat pads with high IL-6-producing breast cancer cells and the depletion of MDSCs by an anti-Gr1 antibody reduced the numbers of metastatic nodules in the lung. Moreover, it was shown that when MDSCs were exposed to conditioned media of metastasizing cancer cells, MDSCs secreted exaggerated IL-6 and soluble IL-6Rα, which induced persistent activation of STAT3 in cancer cells. ADAM family proteases responsible for the shedding of IL-6Rα were also increased on metastasizing cancer-expanded MDSCs. Furthermore, we confirmed that IL-6 trans-signaling was an important mechanism supported by tumor-infiltrating MDSCs to drive the metastatic behavior of cancer cells *in vitro *and *in vivo*.

## Abbreviations

Adam: a disintegrin and metalloprotease; BA: bovine serum albumin; CM: conditioned media; CTL: cytotoxic T cell; ELISA: enzyme-linked immunosorbent assay; EMT: epithelial-to-mesenchymal transition; FACS: fluorescence-activated cell sorting; H & E: hematoxylin and eosin; IL-6: interleukin-6; MDSC: myeloid-derived suppressor cells; NOG: NOD/scid/IL-2Rγ chain-deficient; PBS: phosphate-buffered saline; PCR: polymerase chain reaction; PFA: paraformaldehyde; shRNA: short hairpin RNA; STAT3: signal transducer and activator of transcription 3; TCR: T cell receptor.

## Competing interests

The authors declare that they have no competing interests.

## Authors' contributions

KO designed the research, performed the experiments, interpreted the data and wrote the manuscript; **OYL **performed most of the experiments; **SYS**, **ON **and **PMR **conducted RT-PCR and immunohistochemistry experiments; **MWS **generated the animal models; **DSL **designed the research, interpreted the data, wrote and edited the manuscript. All authors read and approved the final manuscript.

## Supplementary Material

Additional file 1 **Figure S1**. IL-6 levels in culture supernatants of cancer cells, as determined by ELISA. **Figure S2**. EMT6 and 4T1 cells were injected into the mammary fat pads of BALB/c mice. The percentages of MDSCs (CD11b^+^Gr-1^+^) at 19 days were analyzed by flow cytometry (*n *= 4). **Figure S3. (A) **IL-6 was overexpressed on EMT6 cells and IL-6 levels in culture supernatant of selected clone was determined by ELISA. **(B) **EMT6_Con and EMT6_IL-6 cells were injected into the mammary fat pads of BALB/c mice. MDSCs were analyzed at 21 days (*n *= 6). **Figure S4**. Splenic MDSCs from naïve and tumor-bearing mice were treated with EMT6-CM, 4T1-CM, or recombinant IL-6 (1 ng/ml) for 6 hours. IL-6 mRNA expression in 4T1-CM-treated splenic MDSCs was detected by RT-PCR. **Figure S5**. Splenic MDSCs from 4T1 cell-bearing mice treated with 4T1-CM for the indicated periods of time. Signaling molecules were detected by Western blotting. **Figure S6**. Immunofluorescence staining of Gr-1 (red) and IL-6 (green) in the spleen and tumor of EMT6 cell-bearing mice. Scale bar = 30 μm (original magnification, ×1,000). **Figure S7**. 4T1 cells were treated with 4T1-CM for the indicated periods of time. Phosphorylated STAT3 were detected by Western blotting. **Figure S8**. Soluble IL-6Rα levels in culture supernatants of 4T1 cells were measured by ELISA. **Figure S9**. TAPI-2 (100 μM) or Protease inhibitors cocktail (3x) were applied to cultures of splenic MDSCs from 4T1 cell-bearing mice for 18 hours. **(A) **Membrane-bound IL-6Rα was detected by FACS and **(B) **soluble IL-6Rα levels were measured by ELISA. **Figure S10. (A) **Immunofluorescence staining of Gr-1 (red) and IL-6Rα (green) in spleen, tumor, and lung tissues from 4T1 cell-bearing mice. **(B) **Immunofluorescence staining of Gr-1 (red) and IL-6Rα (green) in spleen from EMT6 cell-bearing mice. **(C) **Immunofluorescence staining of Gr-1 (red) and Adam17 (green) in spleen from 4T1- and EMT6 cell-bearing mice. Scale bar = 30 μm. **Figure S11**. Stat3-knockdown 4T1 cells (4T1_shSTAT3 cells) generated using the lentiviral vector containing the short hairpin RNA. STAT3 expression was detected by Western blotting assay. **Figure S12**. Click here for file
