# Supplementary material for: A mutual activation loop between breast cancer cells and myeloid-derived suppressor cells facilitates spontaneous metastasis through IL-6 trans-signaling in a murine model
Source: Breast Cancer Res. 2013 Sep 10;15(5):R79. doi: 10.1186/bcr3473 (PMC3979084; doi:10.1186/bcr3473)

**Figure S1**

**
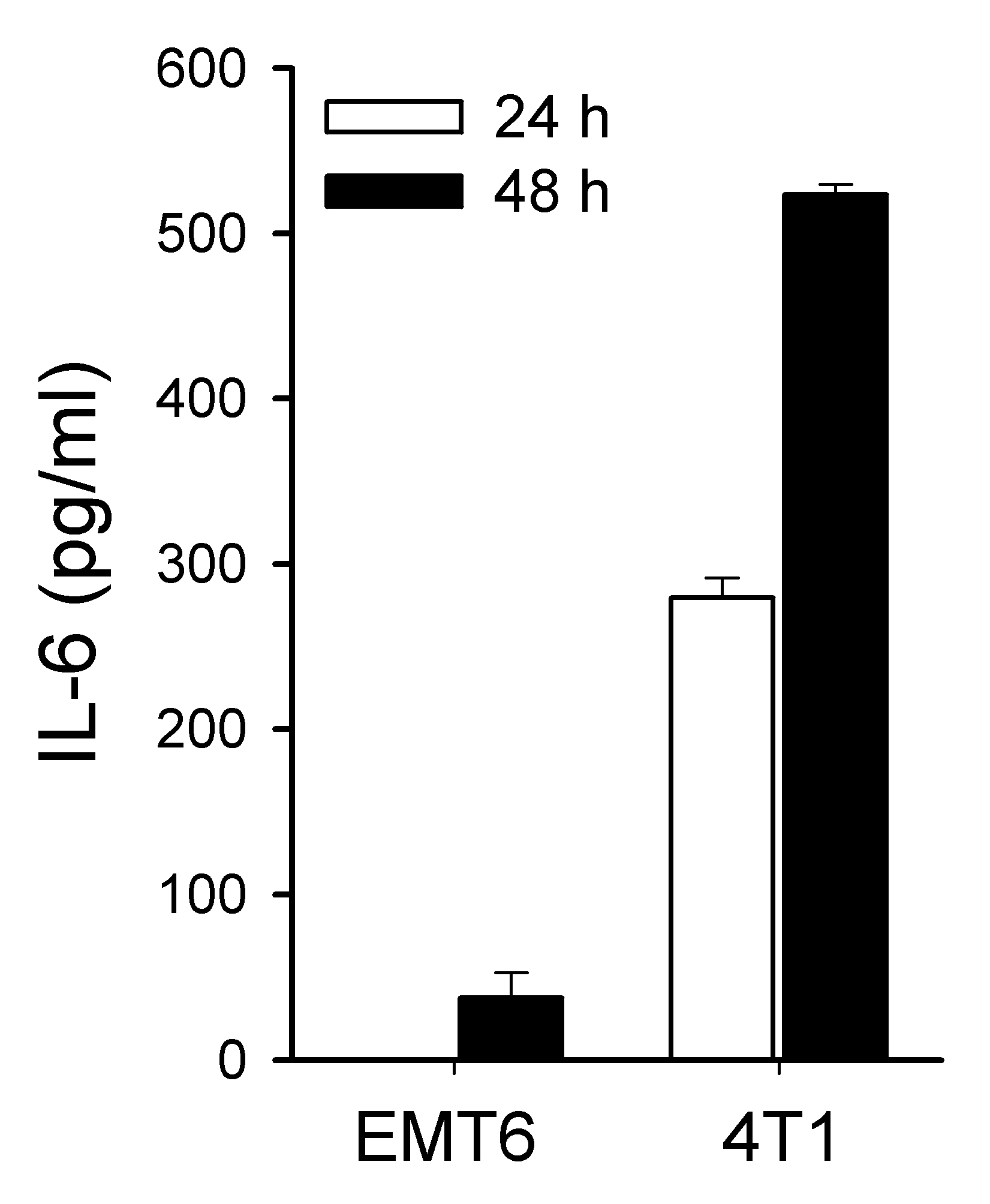
**

**Figure S2**


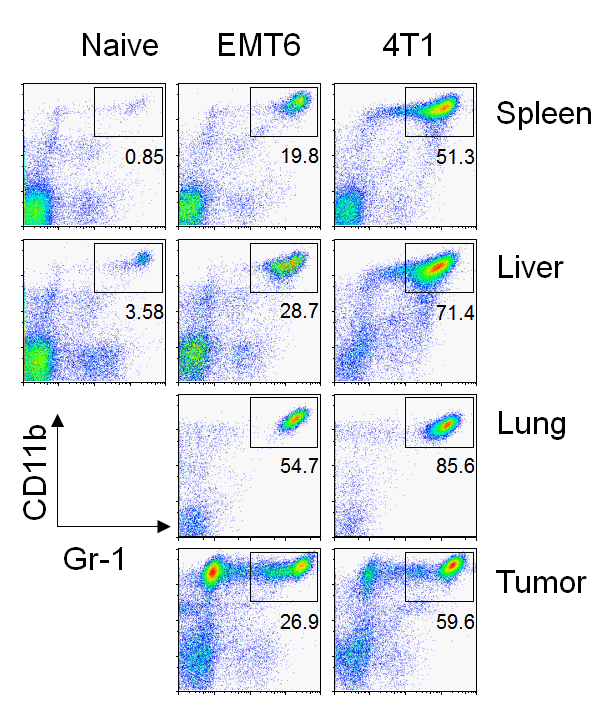


**Figure S3**


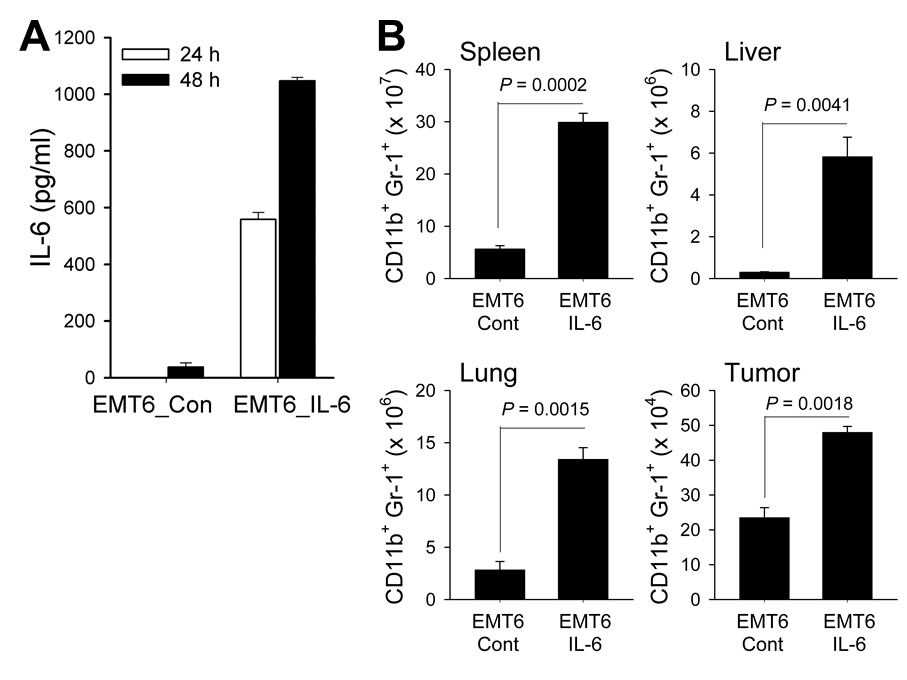


**Figure S4**

**
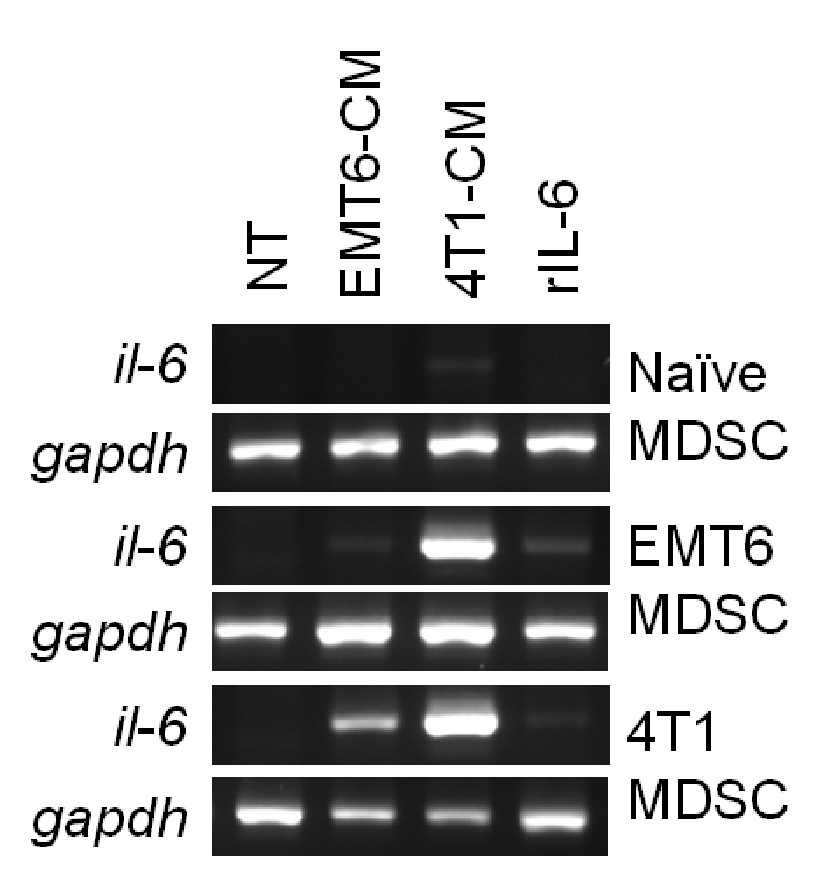
**

**Figure S5**


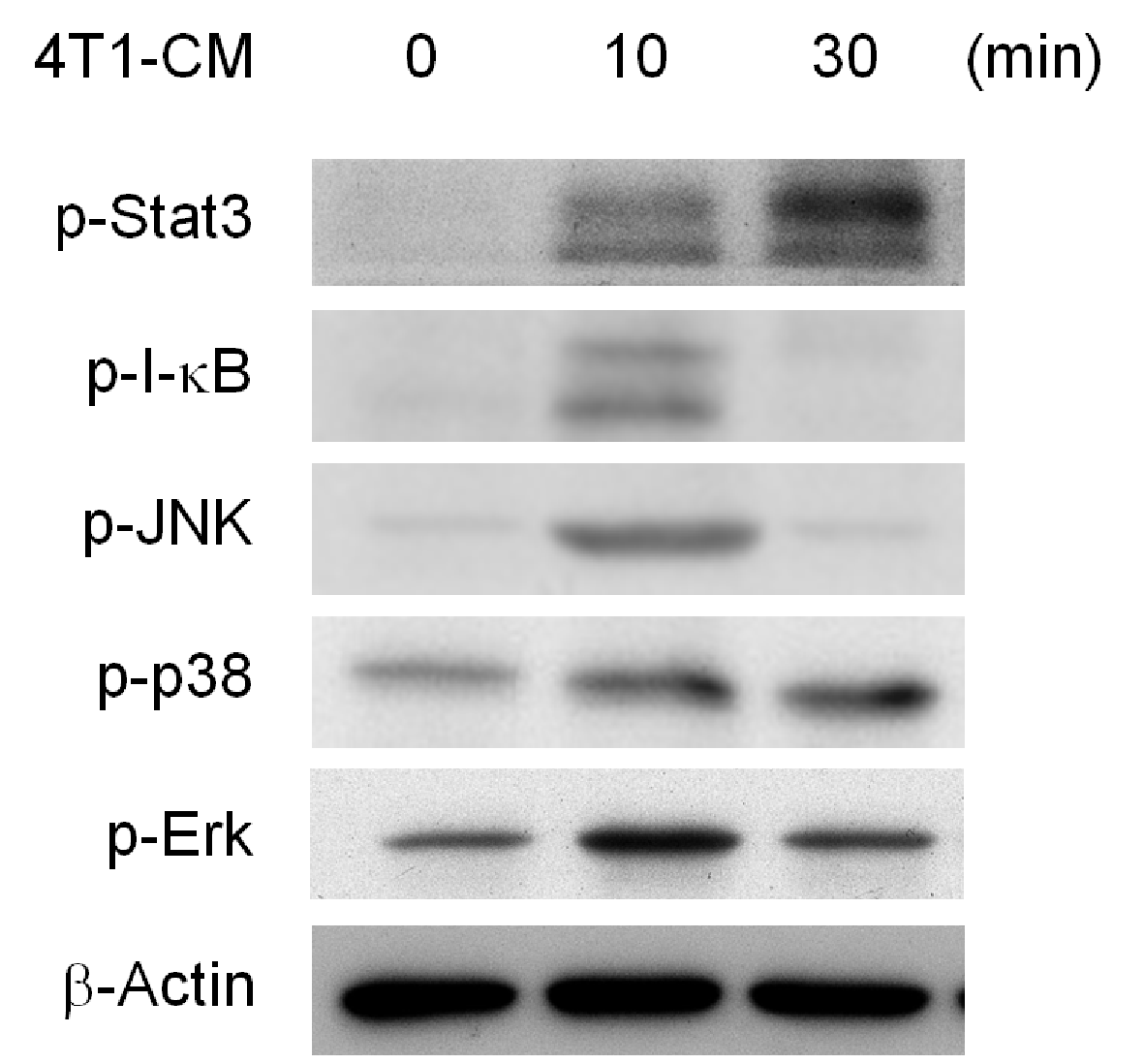


**Figure S6**


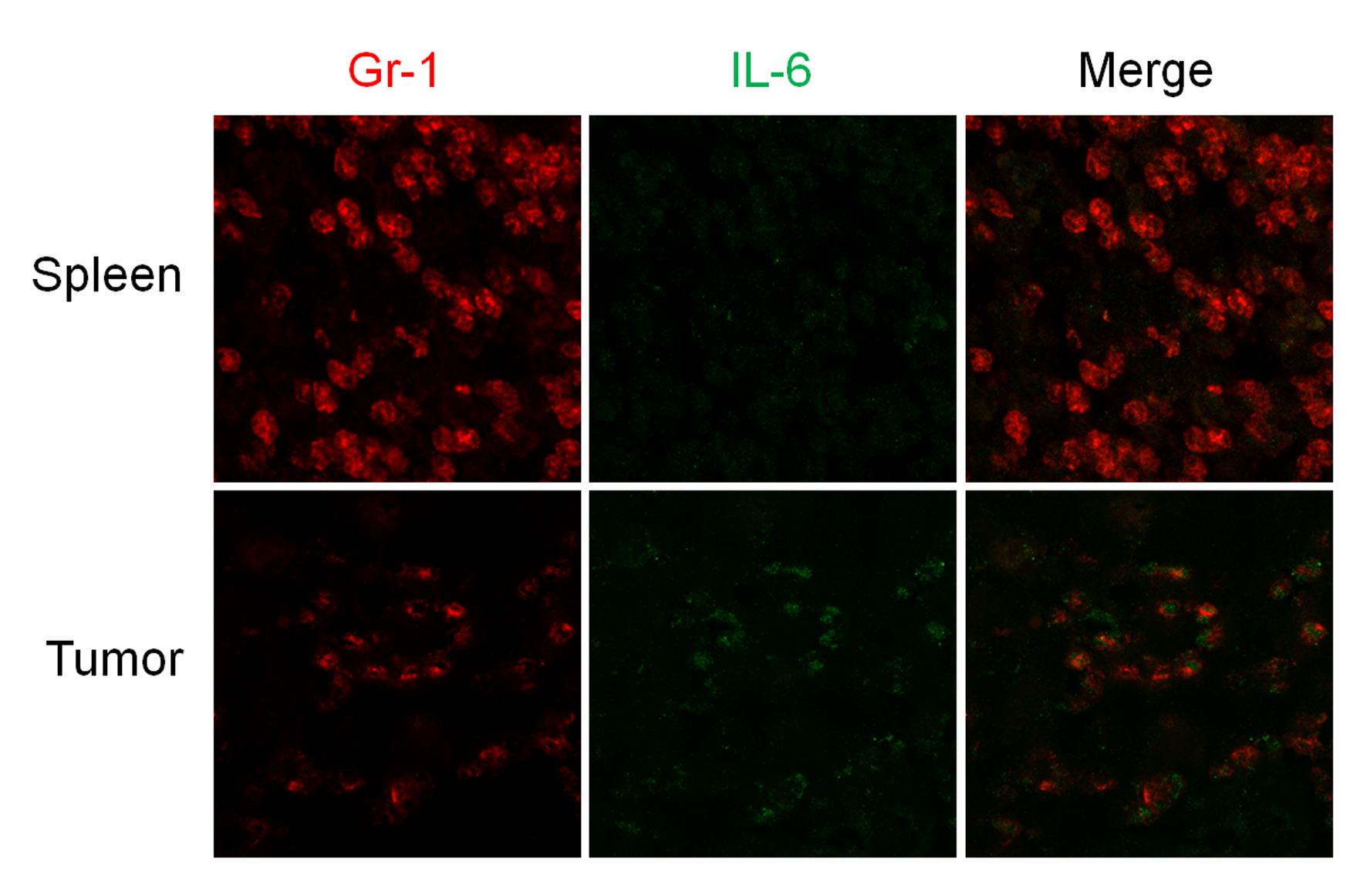


**Figure S7**


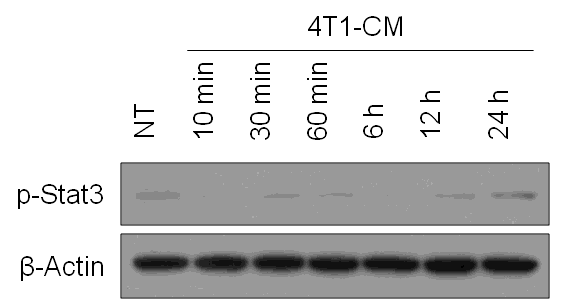


**Figure S8**


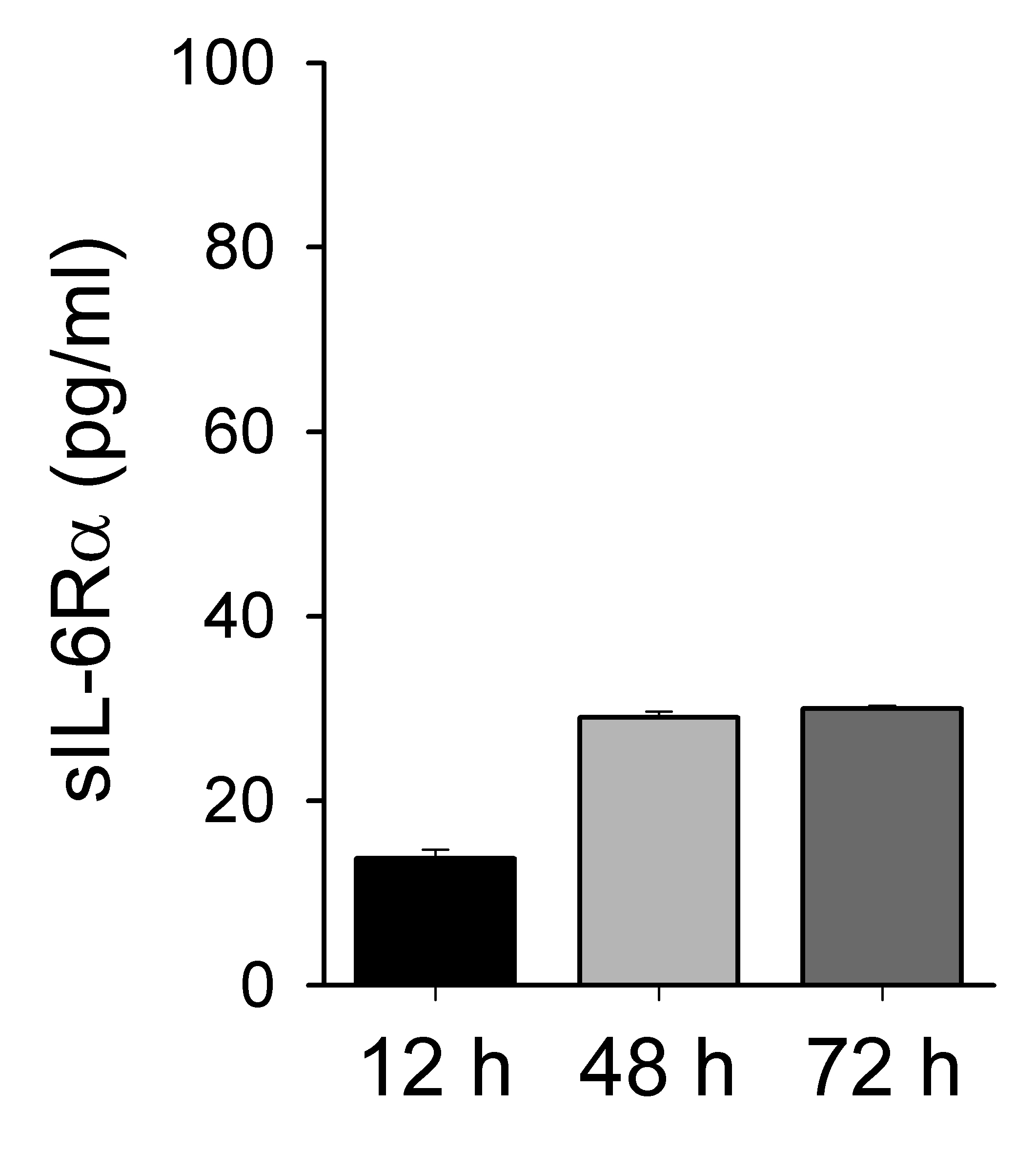


**Figure S9**

**
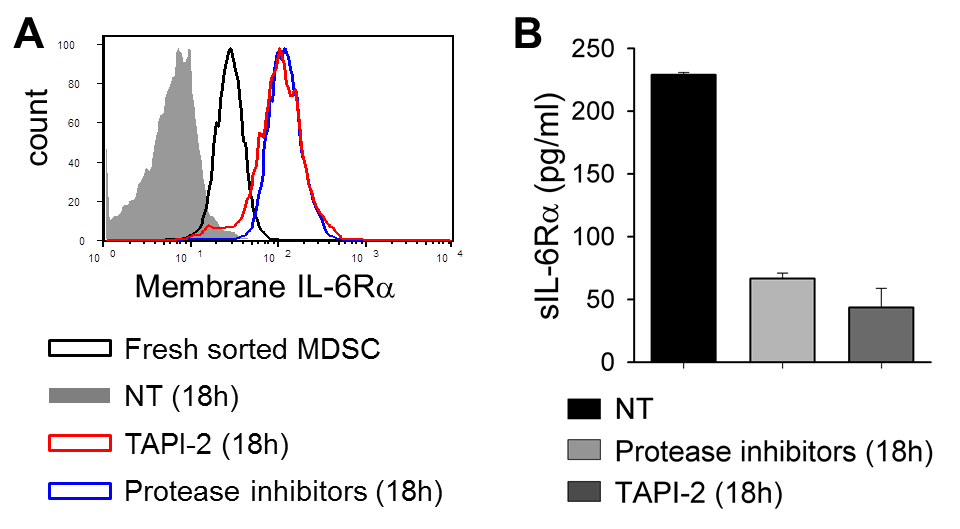
**

**Figure S10**


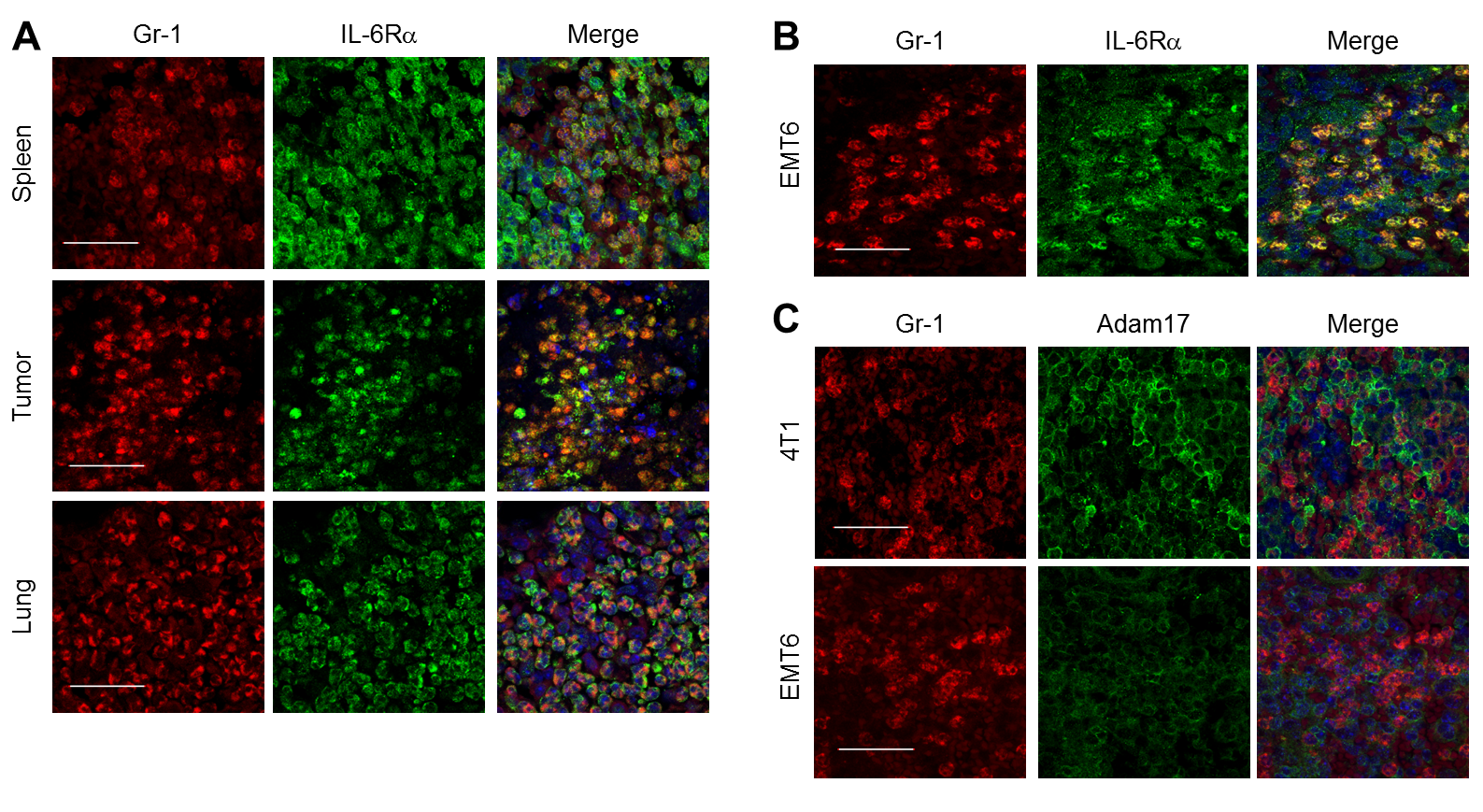


**Figure S11**


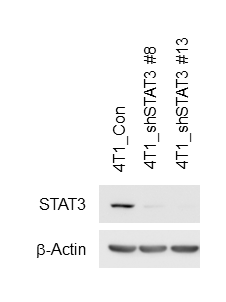


**Figure S12**


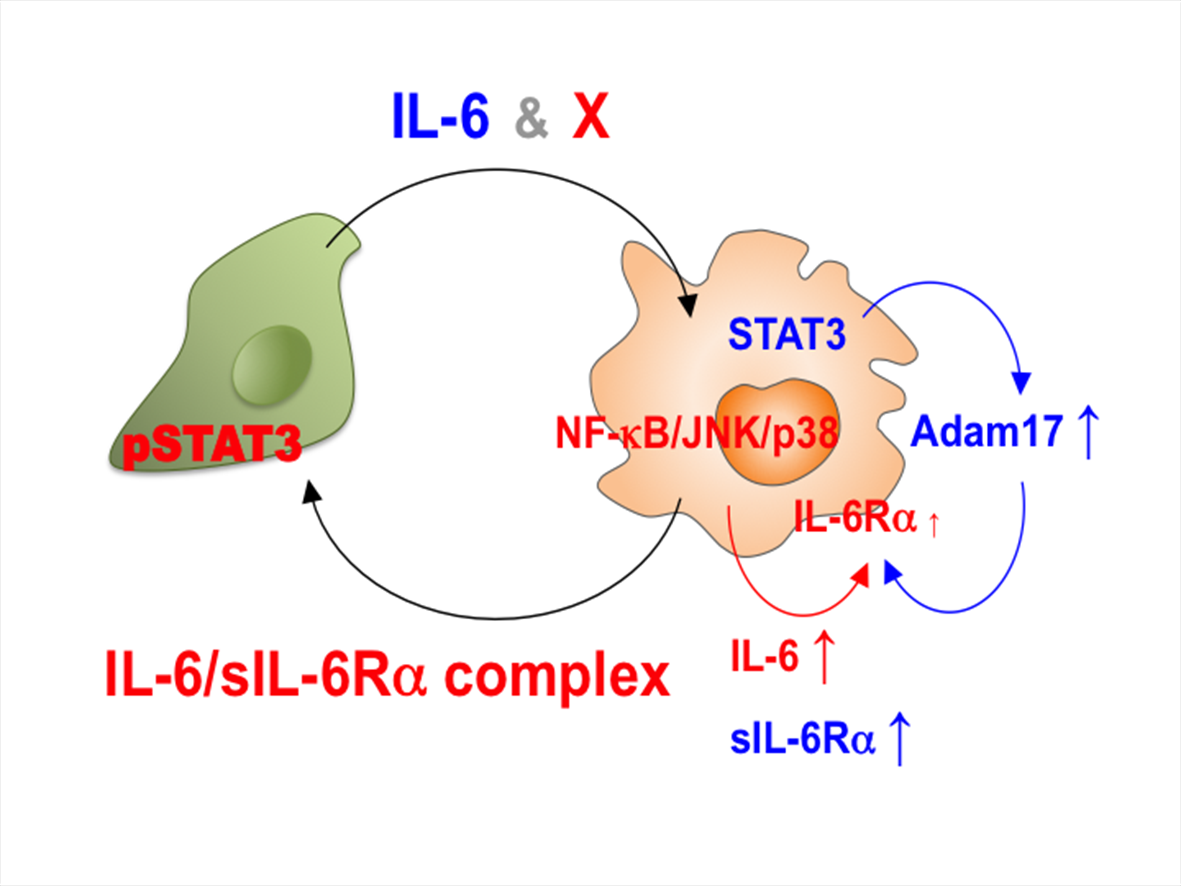

Supplement: Additional file 1 — Figure S1. IL-6 levels in culture supernatants of cancer cells, as determined by ELISA. Figure S2. EMT6 and 4T1 cells were injected into the mammary fat pads of BALB/c mice. The percentages of MDSCs (CD11b+Gr-1+) at 19 days were analyzed by flow cytometry (n = 4). Figure S3. (A) IL-6 was overexpressed on EMT6 cells and IL-6 levels in culture supernatant of selected clone was determined by ELISA. (B) EMT6_Con and EMT6_IL-6 cells were injected into the mammary fat pads of BALB/c mice. MDSCs were analyzed at 21 days (n = 6). Figure S4. Splenic MDSCs from naïve and tumor-bearing mice were treated with EMT6-CM, 4T1-CM, or recombinant IL-6 (1 ng/ml) for 6 hours. IL-6 mRNA expression in 4T1-CM-treated splenic MDSCs was detected by RT-PCR. Figure S5. Splenic MDSCs from 4T1 cell-bearing mice treated with 4T1-CM for the indicated periods of time. Signaling molecules were detected by Western blotting. Figure S6. Immunofluorescence staining of Gr-1 (red) and IL-6 (green) in the spleen and tumor of EMT6 cell-bearing mice. Scale bar = 30 μm (original magnification, ×1,000). Figure S7. 4T1 cells were treated with 4T1-CM for the indicated periods of time. Phosphorylated STAT3 were detected by Western blotting. Figure S8. Soluble IL-6Rα levels in culture supernatants of 4T1 cells were measured by ELISA. Figure S9. TAPI-2 (100 μM) or Protease inhibitors cocktail (3x) were applied to cultures of splenic MDSCs from 4T1 cell-bearing mice for 18 hours. (A) Membrane-bound IL-6Rα was detected by FACS and (B) soluble IL-6Rα levels were measured by ELISA. Figure S10. (A) Immunofluorescence staining of Gr-1 (red) and IL-6Rα (green) in spleen, tumor, and lung tissues from 4T1 cell-bearing mice. (B) Immunofluorescence staining of Gr-1 (red) and IL-6Rα (green) in spleen from EMT6 cell-bearing mice. (C) Immunofluorescence staining of Gr-1 (red) and Adam17 (green) in spleen from 4T1- and EMT6 cell-bearing mice. Scale bar = 30 μm. Figure S11. Stat3-knockdown 4T1 cells (4T1_shSTAT3 cells) generated [file bcr3473-S1.DOC]
